# Supplementary material for: Low-frequency tibial neuromodulation excites bladder activity in humans
Source: eLife. 2026 Sep 4;14:RP106174. doi: 10.7554/eLife.106174 (PMC13544907; doi:10.7554/eLife.106174)
Supplement: Supplementary file 1 [file elife-106174-fig4-data1.pdf]

Appendix B – Urge Intensity Survey (For Printing)

Urge Intensity Survey 1

Please select one of the following options that best describes how intense your current urge to urinate is.

0 - Convenience (No Urge)	
1 – Mild Urge (Can Hold for Greater Than One Hour)	
2 – Moderate Urge (Can Hold for 10 – 60 Minutes)	
3 – Severe Urge (Can Hold for Less Than 10 Minutes)	
4 – Desperate Urge (Must Go Immediately)	

Urge Intensity Survey 2

Please select one of the following options that best describes how intense your current urge to urinate is.

0 - Convenience (No Urge)	
1 – Mild Urge (Can Hold for Greater Than One Hour)	
2 – Moderate Urge (Can Hold for 10 – 60 Minutes)	
3 – Severe Urge (Can Hold for Less Than 10 Minutes)	
4 – Desperate Urge (Must Go Immediately)	
