# Supplementary material for: Low-frequency tibial neuromodulation excites bladder activity in humans
Source: eLife. 2026 Sep 4;14:RP106174. doi: 10.7554/eLife.106174 (PMC13544907; doi:10.7554/eLife.106174)
Supplement: Peer review history

# Editor report: eLife Assessment

**DOI:** 10.7554/eLife.106174.3.sa0

**Authors:** Hayriye Cagnan

**Affiliations:** Imperial College United Kingdom

**Keywords:** Important, Solid

This **important** study investigates frequency-dependent effects of transcutaneous tibial nerve stimulation (TTNS) on bladder function in healthy humans and, through a computational model, shows that low-frequency stimulation accelerates, and high-frequency delays, the urge to void. The integration of experimental and modeling approaches provides a **solid** proof-of-concept foundation for clinical trials targeting urinary retention. However, concerns were raised about over-interpretation of modest effects and the limited physiological validity of the computational model, and the need for replication in clinical populations. Some conclusions, particularly in the abstract, could be further tempered to better align with the strength of the available evidence.

# Referee report: Reviewer #1 (Public review)

**DOI:** 10.7554/eLife.106174.3.sa1

Summary:

This manuscript examines the frequency-dependent effects of transcutaneous tibial nerve stimulation (TTNS) on bladder function in healthy volunteers, supported by a conductance-based computational model of lower urinary tract (LUT) neural circuitry. The authors show that 1 Hz TTNS modestly hastens the urge to void, while 20 Hz TTNS delays it - a finding with potential therapeutic relevance for underactive bladder (UAB). A computational model incorporating spinal, brainstem, and peripheral circuit elements provides a mechanistic framework suggesting brainstem-mediated pathways underlie these frequency-dependent effects. The revised manuscript addresses the majority of concerns raised in the initial review.

Strengths:

Novelty. Demonstrating a low-frequency excitatory effect of TTNS in humans is genuinely new. The possibility of inverting the therapeutic effect of an established neuromodulation intervention by simply adjusting stimulation frequency is clinically meaningful and opens a plausible treatment avenue for UAB.

Integrated approach. Combining a controlled human pilot study with a systems-level neural model is a notable strength. The model is physiologically grounded and serves well as a proof-of-concept tool for exploring mechanistic hypotheses.

Improved reproducibility. The addition of a public GitHub repository with documented code, supplementary figures detailing electrode placement and stimulation parameters, and removal of the externally derived Figure 3 all meaningfully improve transparency.

Improved statistics. The shift to Bayesian modelling with ROPE analysis is well-justified given the small sample size and more appropriate than frequentist testing in this context.

Improved presentation. Unit standardization, figure label corrections, and replacement of imprecise terminology (e.g., "paradoxical", "analytically") make the revised manuscript considerably clearer.

Remaining Concerns:

Afferent-efferent disconnect. The human study measures urgency (an afferent sensory endpoint), while the model's primary output is contraction duration (an efferent motor endpoint). The authors have added discussion of this mismatch, but should state more explicitly that the two lines of evidence are complementary rather than directly comparable, and that the mechanistic link between them remains a hypothesis.

Clinical contextualization of effect size. The excitatory effect of 1 Hz TTNS is modest. A brief reference to what a minimally clinically important difference might look like in UAB or urodynamics research would help readers gauge the translational significance of the finding.

Overall Appraisal:

The authors have achieved their stated aims: providing proof-of-concept human evidence for frequency-dependent TTNS effects and a plausible neural circuit explanation. The manuscript is now appropriately cautious in its claims. The open-source computational model is a useful community resource. This work is best understood as a well-scoped proof-of-concept study that credibly motivates further investigation.

# Referee report: Reviewer #2 (Public review)

**DOI:** 10.7554/eLife.106174.3.sa2

Strengths:

The main strength of the work is to call attention to a new possibility of inverting the effect of TNS in humans by manipulating stimulation frequency, opening new indications for the therapy. This is highly relevant because of the recent popularity of TNS and its non-invasiveness, which lends itself to rapid testing and evaluation for new conditions and high willingness to adopt. The authors convincingly demonstrate a modest excitatory effect on bladder sensation with low-frequency TNS, which clearly warrants further investigation.

The high-level design of the hypotheses, concepts, and experiments are clearly articulated in both the methods and in particularly clear diagrams, letting the reader focus their attention on the most important findings.

It is rare to develop a new computational model of the lower urinary tract at a systems level, and even more so for it to incorporate circuits in the spinal cord and brainstem centers, and this work undoubtedly advances the field's ability to engineer such systems. Further, because the model is comprised of linked conductance-based point-neurons, it is an excellent tool to investigate how an arguably plausible wiring diagram for neural control of the LUT could result in stimulation frequency dependent effects on pelvic efferents. It is a proof of concept demonstrating how their mechanistic hypothesis of TNS could be implemented neurophysiologically by the nervous system. Further, the model is shared openly, which conforms to good modeling practices.

Weaknesses:

The main drawback of the work is the overinterpretation of the results. The human study and computational model are both proof-of-principle. The human study effect size is small and the sample size is modest; the computational model is poorly validated and does not generate physiologically typical urodynamic responses when simulating even healthy nominal LUT conditions. Thus, both the existence of a TNS 1Hz inhibitory effect (human study) and the mechanistic interpretation of its origin (simulations) remain provisional. For example, despite some caveats later in the work, the abstract stating there is a "frequency-dependent effect of TNS via the ability to alter urge perception and down-regulate bladder activity, corroborating model predictions," could easily be misleading, since (a) the reduction in time of first urge with 1Hz stimulation was quite small relative to overall void time, (b) reported intensity was essentially not impacted, and (c) the model does not directly make predictions about these experiment outcome measures. Similar overreaching statements appear in the second to last paragraph of the introduction, the first paragraph of the discussion, and so on throughout the paper. Many of the analyses are bespoke to the idiosyncrasies of the dataset rather than field standards, making spurious results also more likely and the effects provisional. One example is the use of robust linear regression to identify significance in the experiment between the 1Hz and control groups AND removing outliers before the analysis, since the typical approach is to use robust regression when the outliers are left in the data. Taken together, the potential excitatory effect and mechanism are interesting, and perhaps worth further investigation, but are considerably more tentative than stated.

It remains ambiguous whether a TNS excitatory effect size shown (even if it ends up being repeatable) is clinically meaningful. The ROPE analysis is a reasonable start, but no attempt to connect the parameters chosen (e.g. 60s) to clinical outcomes were made. This is especially true given the washout results and lack of effect on perceived urgency.

There remain several reasons to treat the model results questionable. First, as the authors now note, the model under normal conditions does not generate normal function; a voiding efficiency of 15% is severely underactive. Second, the 1 Hz stimulation simulation appears to create normal voiding, suggesting that the implementation of the neural control circuits may not produce results that would generalize to other experiments. Third, analysis focuses on the model outcome of "time to void", but this outcome is not reported for the experiment, so direct comparison is not possible.

# Author comment: Author response

**DOI:** 10.7554/eLife.106174.3.sa3

**Authors:** Aidan McConnell-Trevillion, Milad Jabbari, Wei Ju, Elliot Lister, Abbas Erfanian, Srinjoy Mitra, Kianoush Nazarpour

**Affiliations:** University of Edinburgh Edinburgh United Kingdom; University of Edinburgh Edinburgh United Kingdom; University of Edinburgh Edinburgh United Kingdom; University of Edinburgh Edinburgh United Kingdom; Iran University of Science and Technology Tehran Islamic Republic of Iran; University of Edinburgh Edinburgh United Kingdom; University of Edinburgh Edinburgh United Kingdom

The following is the authors’ response to the original reviews.

**Public Reviews:**

**Reviewer #1 (Public review):**

Summary:

The research investigates the frequency-dependent effects of transcutaneous tibial nerve stimulation (TTNS) on bladder function in healthy humans and via a computational model. The authors report that low-frequency (1 Hz) TTNS accelerates the urge to void, while highfrequency (20 Hz) TTNS delays it, corroborated by a computational model suggesting brainstem-mediated mechanisms. The work bridges experimental and theoretical approaches to propose a novel framework for TTNS applications in urinary retention.

Strengths:

(1) The integration of human experiments and computational modeling is a major strength. The model successfully replicates bladder dynamics and provides mechanistic insights into frequency-dependent effects.

(2) Identifies potential therapeutic applications for urinary retention, a condition with limited non-invasive treatments.

(3) Figures are clear and illustrative, and supplementary materials provide essential methodological depth.

(4) Controlled experimental design (eg., single-blinded, fluid/caffeine restrictions, etc), detailed computational model parameters and validation against animal data, transparency in data exclusion criteria and statistical adjustments.

Weaknesses:

(1) The study uses healthy participants; extrapolation to clinical populations (e.g., urinary retention patients) requires validation.

The authors have included a statement noting this and explaining that future work will explore this.

(2) The simulated bladder capacity (100-150 mL) is lower than physiological ranges (300400 mL). While the authors note this, the impact on model validity should be further addressed.

The authors acknowledge that the simulated bladder capacity and voiding efficiency of the model are lower than human physiological ranges. They have added an additional explanatory paragraph detailing this limitation and proposing the animal training data as a possible cause. Despite these limitations we do not believe this prevents the model from being used to explore proof-of-concept hypotheses (e.g., presence of frequency dependence, potential mechanistic bases) as in the present paper.

(3) The model omits nociceptive afferents, limiting its applicability to pathological conditions like overactive bladder.

The authors acknowledge that this is a limitation of the model, and have included a paragraph in the paper’s discussion detailing the limited scope of our *in silico* approach and clarifying the extent to which the results may be interpreted.

(4) The lack of significant differences in urge intensity between groups (despite timing differences) warrants deeper discussion. Is the primary effect on efferent activity (as suggested) rather than sensory perception?

The authors acknowledge that this is a surprising result and as such have deepened the discussion of the pilot study results, including hypothesizing as to potential explanations and suggesting further research in the area.

(5) One of the highlights of this study is the identification of the effect of low-frequency (1 Hz) tibial nerve stimulation (TNS) on facilitating bladder contraction. Although the authors have clarified this effect in healthy participants, it would strengthen the conclusion if a UAB animal model (e.g., PMCID: PMC7927909, PMC8163611, PMC7847056, PMC8799394) were used to evaluate the same effect.

The use of animal models is out with the scope of this study which aimed to act as a proof of concept work using a primarily computational approach backed by preliminary human data. The authors acknowledge that this does limit the strength of the conclusions. However, several animal models have been utilized in previous work (as cited in the publication) that demonstrate an excitatory effect of low-frequency tibial nerve stimulation. This work builds upon these previous studies to strengthen the case for a frequency dependent effect of the intervention.

**Reviewer #2 (Public review):**

Summary:

Tibial nerve (electrical) stimulation (TNS) has emerged over the past 15 years as a non-invasive method to treat bladder overactivity, but interestingly, new animal work has suggested that TNS could actually be used to excite the bladder when appropriately tuning the stimulation frequency, effectively inverting its effect, perhaps opening the door to treat different conditions (e.g., UAB). The present study tests how healthy people respond to low and high frequency TNS, with the authors showing that they can substantially delay people's first sensation of bladder fullness with high frequencies (20Hz, shown many times before) but also that they can slightly hasten people's first sensation with low frequencies (1Hz, new result in humans). Moreover, the authors develop a computational model of interconnected conductance-based simulated neurons arranged in a physiologically plausible circuit that reproduces some aspects of the frequency-dependent effects of TNS. Their simulations suggest that we might expect low-frequency TNS to also increase the duration of bladder contractions in humans. The study highlights a potential new research direction, optimizing TNS stimulation parameters to increase basal bladder excitability.

Strengths:

The main strength of the work is to call attention to a new possibility of inverting the effect of TNS in humans by manipulating stimulation frequency, opening new indications for the therapy. This is highly relevant because of the recent popularity of TNS and its non-invasiveness, which lends itself to rapid testing and evaluation for new conditions and a high willingness to adopt. The authors convincingly demonstrate a modest excitatory effect on bladder sensation with low-frequency TNS, which clearly warrants further investigation.

The high-level design of the hypotheses, concepts, and experiments is clearly articulated in both the methods and in particularly clear diagrams, letting the reader focus their attention on the most important findings.

It is rare to develop a new computational model of the lower urinary tract at a systems level, and even more so for it to incorporate circuits in the spinal cord and brainstem centers, and this work undoubtedly advances the field's ability to engineer such systems. Further, because the model is comprised of linked conductance-based point-neurons, it is an excellent tool to investigate how an arguably plausible wiring diagram for neural control of the LUT could result in stimulation frequency-dependent effects on pelvic efferents. It is a proof of concept demonstrating how their mechanistic hypothesis of TNS could be implemented neurophysiologically by the nervous system.

Weaknesses:

The main drawback of the work is the frequent over-interpretation of the results. The human study and computational model are both proof-of-principle studies because the experimental effect size and sample size are modest, and the computational model is poorly validated and does not generate physiologically typical cystometric responses in simulations that are designed to recapitulate nominal LUT behavior.

Despite the stated caveats about the small effect in the human study, it should be emphasized throughout that this result is most reasonably interpreted as showing the possibility that TNS can have a low-frequency excitatory effect that merits follow-up, rather than a conclusive demonstration. The effect size is small (as the authors note) and should be placed in context with some minimally clinically important difference, if possible. The result is statistically significant, but even this may be subject to revision due to the small sample and the effect of post-hoc outlier removal and data analysis choices.

Acknowledged, the authors have included caveats in the discussion making clear that the present results should be interpreted as a proof of concept rather than a definitive demonstration. We note that in combination with existing animal findings these results strengthen the case for the existence of an unexplored excitatory effect of TTNS in human beings that may have valuable clinical implications if generalised.

Given the apparent mismatch between the model and the cystometric behavior at the systems level in the "normal" case (e.g., low capacity, low voiding efficiency, omitted pressure profiles, frequency, etc.) and the absence of quantitative model validation (e.g., it was not compared directly with any experimental data from human urodynamics or rodent cystometry, beyond the initial fit to the neural data, no sensitivity analyses were performed, no goodness of fit computed, etc.) the discussion should be much more circumspect about interpreting the results at a systems level and should probably contain a paragraph explicitly detailing the limitations of the model. The subsequent interpretation should focus narrowly on the neural circuitry, rather than things like contraction duration, where the model is at its strongest. As written, the authors over-interpret what the in silico study can reasonably be used to infer about LUT function.

The authors have reworded the discussion section, including a limitations paragraph containing caveats about the interpretation of the results. We make clear that a systemslevel perspective should be maintained and that futher research is required to validate and generalise these results.

More justification is needed for why the contraction duration of the model is the central focus of analysis, when it connects only tentatively to the human study results, which focus on urgency. While not necessarily incorrect, a clearer link or motivation should be offered for how this informs our understanding of frequency-dependent TNS afferent or efferent inhibition during filling (which was the focus of the human studies and the abstract). In other words, why doesn't the model reproduce the 1Hz excitation effect of expediting void onset (or urgency in the human study), and why is it justified to look at contraction duration as a surrogate measure?

The authors acknowledge this issue, and have included an additional section to the discussion considering the disparity between afferent and efferent effects observed across the pilot study and computational experimentation. The need for further research within this area to disentangle the complex nature of the frequency dependence has been stressed.

The authors claim that "voiding behavior occurred earlier [at 1Hz stim in the model]", pointing to Figure 6A as evidence, but this panel appears to show a single example model run where 1Hz voiding occurs only ~1s earlier (display makes this very hard to estimate). This is insufficient evidence to support the claim. Later, it is stated that "TNS did not ... void much earlier". The claims should be made compatible, and all such claims should have reasonable supporting evidence.

The authors have included additional information in the supplementary materials to support the claim.

This information includes the bladder volume profile of a number of simulations under 0Hz and 1Hz conditions as well as the average void-onset time (i.e., simulated time before first void).

There are a number of reporting concerns that can be easily addressed:

(1) Human Study:

(a) To interpret the human study analysis, a fuller description of the "optional 10m inute extension" is necessary. How were participants presented with this option, how was blinding preserved, what fraction of participants accepted, and did phase 1 results influence their decisions to continue?

The authors have included additional clarification detailing how blinding was maintained during the washout period. Additionally, we have included a section in the results which details participation rates for the washout period. Given that only one participant declined participation in the washout period we do not believe it is necessary to conduct an analysis on what factors influenced participation.

(b) For reproducibility, details about the TNS parameters should be articulated, such as the method of determining "motor thresholds" (unless this is synonymous with "urge to urinate"), the shape of the stimulation pulses (e.g., biphasic, charge balanced), typical applied current, etc.

The authors have included the requested information and added two figures to the supplementary materials detailing the parameters of the equipment and the exact electrode placement used during the pilot study.

(2) The Computational Model

(a) The code availability statement for this type of work is inadequate. The model used for simulations in this work, as well as the code used to initialize (and randomize synaptic connections), needs to be hosted publicly because (i) a model this intricate is extremely hard to reproduce/verify without code, (ii) simulations are an essential piece of the argument, (iii) hosting code requires very little overhead. Although there is an appropriate level of detail in the model description, it would not be possible to reproduce the model in any reasonable amount of time (or at all) because of the implementation-level details that are, understandably, omitted from the methods (e.g., what is a "unit", what 'exactly' do the connections in the PMC and PAG diagrams relate to, what were the final parameters used for all conductances, which parameters were "matched" to the original papers and which were not, etc.).

The authors have included a link to a public GitHub repository where any interested individuals may download and use the code on their own machines for their own purposes. The repository, which includes a readme file detailing the operation of the model, as well as the thoroughly documented code provide the necessary transparency as suggested by the reviewers. We hope that by making the code open-source in this manner further research efforts by any interested researchers will be stimulated.

(b) Critical cystometric/urodynamic values that are typically analyzed to assess healthy LUT function are detrusor pressure (timeseries) and/or post-void residual or voiding efficiency (scalars). These should be included to verify that the model is representative of the "normal" case. This is especially important because the model's "normal" behavior appears to have extremely low voiding efficiency (Figure 6A).

The authors acknowledge this limitation and as such have modified the simulation files to calculate and return: detrusor pressure, post-void residual, bladder capacity, and voiding efficiency (calculated post-hoc from these values). It should be noted however, that implementing this change required that the computational results be re-run using the new code. As such, the exact details of Figure 5 now differ slightly (though the high-level results and implications remain unchanged).

While the high-level results surrounding the frequency-dependence of TTNS and the likely brainstem specific cause of this effect remain unchanged, there were minor changes in the results of the computational projection experiments that necessitated a re-write of a portion of the results section.

Additionally, the authors have added a section exploring the low-voiding efficiency of the model at baseline and potential explanatory factors.

**Recommendations for the authors:**

**Reviewer #1 (Recommendations for the authors):**

(1) In Figure 6Cii, the high frequency is labeled as 10 Hz, but it should be 20 Hz. The authors should correct this in the figure legend.

Acknowledged, the typo has been corrected.

**Reviewer #2 (Recommendations for the authors):**

(1) Data and Analysis:

(a) Greater detail on analysis exclusion is warranted. What does it mean to have "greater than normal water intake"? Why was a large "urge duration" grounds for exclusion? Was its threshold set post-hoc, which group was that participant from, and does its inclusion (or not) affect the results of the analysis substantially?

The authors acknowledge the issue of data removal. As such, to address this limitation an alternative analysis was conducted. Rather than frequentist methods, a Bayesian modelling approach and post-hoc ROPE analysis was conducted which included a greater proportion of the dataset (excluding only those who did not undergo neuromodulation, or who directly met the exclusion criteria for the study). This approach was taken as bayesian methods are better suited for smaller sample sizes such as the one utilised in the present work. The ROPE analysis provides additional evidence for a real-world relevance of the effect on bladder function. Though the authors acknowledge that these results are preliminary they hope they will provide initial evidence for the translation of a novel effect of TTNS into human participants.

(b) It is my understanding that Figure 4C is a plot of G1Hz and G20Hz on the horizontal from 4A and G1Hz and G20Hz on the vertical from 4B-"before". Hopefully, this is correct, and perhaps there is some way to state more simply what data are being reported, as it took me some time to understand.

The authors confirm that figure 4C is a representation of data from figure panels A, and B. Thee horizontal axis represents the temporal “"urge onset” and the vertical axis the subjective intensity experienced at this point. To clarify this, the authors adjusted the axis labels to make clear the data being reported. Additional clarification was also added to the figure legend.

(c) The choice of units in Figure 6 makes interpretation harder than it needs to be. Although not SI units, the field commonly reports volume in ml and duration in seconds or minutes (certainly not ms). The horizontal on Figure 6A is especially confusing, since sim cycles are not clearly defined, nor is the reason for the 20ms of them, or if the 1000s of total simulation time means compute-time or simulated time. Is Figure 6A (20ms/cyc)(50000cyc)(1s/1000ms)*(1min/60s) = 16.67 min of simulated time? If so, does the model show >6 voiding events in that time under normal conditions (which probably requires some explanation, since that is unusual)? Later (L216), other terminology of "simulation run" is introduced and further complicates the interpretation of how much simulated time is passing.

Acknowledged, the authors have updated the units used in figures througout the publication to match standard SI notation (Fig 4: M^3^ -> ml, Fig. 5A:M^3^ -> ml, 20ms cycles -> seconds, ms->seconds). Authors have also updated the language used in the figure and the paper to make clear that the figure is referring to 500 seconds (16.67 mins) of simulated time.

(d) It appears that in Figure 6B that a contraction duration of 0ms means no contraction at all - unclear if that is also true for everything below the horizontal dashed line.

(e) Using p-values for analyzing differences between average model outputs (Figure 6C) is not appropriate, since one can run the model as many times as needed, making any negligible effect size statistically significant.

The authors acknowledge that the computational nature of the second analysis limits the statistical tests that may be reasonably applied. As such, they have rewritten the results and discussion section to instead compare mean differences/effect sizes without reliance on p-values specifically.

(2) Clarity and Presentation:

(a) Figure 3 should be removed since it describes an experiment not conducted in this study and whose data was used only for model fitting, not an integral component of the model concept, analysis, or results. A short description and a paper reference are sufficient.

The authors acknowledge this feedback and have removed Figure 3 from the publication. We have instead provided a reference and brief description of the data used to fit the parameters of the model.

(b) L46, based on my understanding, should read something like "...may be a frequency dependent of TTNS, where low frequencies up-regulate bladder activity while higher frequencies downregulate it."

Acknowledged, this section has been reworded to improve clarity.

(c) Generally speaking, there is nothing "paradoxical" about a frequency-dependent response to e-stim, which happens throughout the nervous system and even in the LUT with pudendal sensory stimulation. "Surprising", "useful", "underexplored", etc., are all closer to the authors' meaning.

Acknowledged, the authors have avoided the use of the term paradoxical to better represent the original intent of the research findings.

(d) I am used to "washout" rather than "runoff", but this is a journal style decision, and either is fine.

Acknowledged, the authors have replaced the use of the term runoff with washout and adjusted figure 1 to reflect this change.

(e) L51 "analytically" is a mathematical keyword reserved for closed-form solutions, which is not what the authors actually refer to. Something like "computationally" or "in silico" is closer to their meaning.

Acknowledged

(f) L172 "abnormality" should be "non-normality".

Acknowledged

(g) L148 "Like the original model", presumably referring to Gorski?

Correct, wording has been changed to make this clear.

(h) L208-220 Unclear precisely what is meant by "intensity of the voiding events" or "temporal nature of the cycle".

Acknowledged, the authors have provided additional clarification to avoid confusion.

(i) Figure 6C Is "baseline" the nominal model without stimulation, while the "all connected" is the nominal model with stimulation? And all the rest of the conditions indicate what was cut in silico?

Acknowledged, authors have reworded the figure legend to improve clarity.
